# Virtual reality cue exposure for the relapse prevention of tobacco consumption: a study protocol for a randomized controlled trial

**DOI:** 10.1186/s13063-016-1224-5

**Published:** 2016-02-19

**Authors:** Camille Giovancarli, Eric Malbos, Karine Baumstarck, Nathalie Parola, Marie-Florence Pélissier, Christophe Lançon, Pascal Auquier, Laurent Boyer

**Affiliations:** Department of Psychiatry, La Conception University Hospital, 13005 Marseille, France; Aix-Marseille University, EA 3279 - Public Health, Chronic Diseases and Quality of Life - Research Unit, 13005 Marseille, France

## Abstract

**Background:**

Successful interventions have been developed for smoking cessation, but the success of smoking relapse prevention interventions has been limited. In particular, cognitive behavioural therapy (CBT) has been hampered by a high relapse rate. Because relapses can be due to the presence of conditions associated with tobacco consumption (such as drinking in bars with friends), virtual reality exposure therapy (VRET) can generate synthetic environments that represent risk situations for the patient in the context of relapse prevention. The primary objective of this study is to evaluate the effectiveness of CBT coupled with VRET, in comparison to CBT alone, in the prevention of smoking relapse. The secondary objectives are to assess the impact of CBT coupled with VRET on anxiety, depression, quality of life, self-esteem and addictive comorbidities (such as alcohol, cannabis, and gambling). A third objective examines the feasibility and acceptability of VR use considering elements such as presence, cybersickness and number of patients who complete the VRET program.

**Method/design:**

The present study is a 14-month (2 months of therapy followed by 12 months of follow-up), prospective, comparative, randomized and open clinical trial, involving two parallel groups (CBT coupled with VRET versus CBT alone). The primary outcome is the proportion of individuals with tobacco abstinence at 6 months after the end of the therapy. Abstinence is defined by the total absence of tobacco consumption assessed during a post-test interview and with an apparatus that measures the carbon monoxide levels expired. A total of 60 individuals per group will be included.

**Discussion:**

This study is the first to examine the efficacy of CBT coupled with VRET in the prevention of smoking relapse. Because VRET is simple to use and has a low cost, this interactive therapeutic method might be easily implemented in clinical practice if the study confirms its efficacy.

**Trial registration:**

ClinicalTrials.gov Identifier: NCT02205060 (registered 25 July 2014).

**Electronic supplementary material:**

The online version of this article (doi:10.1186/s13063-016-1224-5) contains supplementary material, which is available to authorized users.

## Background

Tobacco use is the leading cause of preventable morbidity, mortality and health expenses in developed countries [1]. In France, the latest health survey estimated in 2010 an overall prevalence of tobacco use of approximately 28.7 % among individuals older than 15 years old [2], resulting in an estimated $47 billion in related annual health and economic costs [3]. Many successful interventions have been developed for smoking cessation [4], but the efficacy of smoking relapse prevention interventions has been limited [5]. The high rates of relapse after smoking cessation programs have ranged between 40 and 70 %, suggesting the need to incorporate more effective strategies for relapse prevention into such programs [6]. A cognitive behavioural model of relapse was developed by Marlatt and colleagues, considering that relapses to drug use are usually associated with high risk situations characterized by the presence of drug-related stimuli [7]. Several studies have reported that individuals with substance use disorders have physiological and subjective reactions to the presentation of drug-related stimuli, a phenomenon known as cue reactivity [8]. Recent studies have also reported that cue-induced craving does not decrease over an extended period of abstinence and might actually increase with a longer duration of abstinence [9]. In addition to drug-related stimuli, the involvement of abnormal cognitive and motivational processes also contributes to high relapse rates [10]. Two techniques have emerged as potential smoking relapse prevention interventions: cognitive behavioural therapy (CBT) and cue exposure therapy (CET). CBT aims to explain and treat the individual's dysfunctions (for example, cognitive patterns and automatic thoughts). CBT can increase the individual's motivation and can play an important role in relapse prevention through analysis and understanding of this phenomenon [11]. However, a recent meta-analysis reported insufficient evidence to support the use of CBT to prevent relapse, and it advised the examining of alternatives in attempts to teach skills to cope with risk situations [12]. In this sense, CET has been considered an interesting complement to CBT [13]. CET consists of controlled and repeated exposure to drug-related cues, using pictures, photographs or video to reduce craving associated with situations of tobacco consumption. CET could extinguish the association of a response (smoking) to a stimulus (for example, an ashtray, a lighter, or a cigarette pack) [14]. However, these situations are difficult to reconstruct effectively in passive video or images and in the framework of a hospital or an office, thus limiting the efficacy of CET.

Because of the complexity of nicotine cue reactivity involving proximal (lit cigarette, ashtray, lighter), contextual (physical situations such as a party or a bar) and complex (a combination of contextual and proximal cues, such as situations involving social interactions where people are smoking or offered cigarettes) cues, more ecological environments should be proposed than those used in CET [15]. Virtual reality exposure therapy (VRET), which is now used for the treatment of various psychological disorders (phobias and post-traumatic stress disorders) [16, 17] and smoking cessation [17–19], can generate synthetic environments that represent risk situations for the patient in the context of relapse prevention [18]. However, this approach has never been used to prevent relapse in abstinent smokers. VRET offers various advantages for cue exposure, such as controlled environments and complex, dynamic interactive, three-dimensional situations (such as virtual bars, offices and artificial smokers in computerized restaurants) [19]. VRET gradually exposes the patient in the confidentiality of an office to situations considered to pose a high risk of relapse. Finally, VRET allows the therapist to guide the patient in real time, helping him or her to modify emotions and addiction-related cognitions.

To our knowledge, CBT coupled with VRET has not yet been evaluated in preventing smoking relapse in subjects with smoking abstinence. The primary objective of this study is to evaluate the effectiveness of CBT coupled with VRET compared to CBT alone in the prevention of smoking relapse. The secondary objectives are to assess the impact of CBT coupled with VRET on dependence, craving and addictive comorbidities (such as alcohol or cannabis). In addition, because smoking abstinence can be associated with negative effects known to influence craving [20, 21], we also explored the effects of VRET on psychological and emotional features, including depression, anxiety, self-esteem and quality of life.

A third objective examines the feasibility and acceptability of VR use. Three main issues will be considered: presence, cybersickness and number of patients who complete the VRET program. Presence is an important issue to consider when investigating the impact of VRET. Presence, defined as a psychological measure of “being there” or in the virtual environment, may be considered as a condition of VRET success [22]. The measure of cybersickness is of the utmost importance when examining feasibility and acceptability [23]. Cybersickness is a natural physiological response to unusual stimuli, which results from an asynchrony between visual, vestibular and proprioceptive information. The subsequent incongruence may produce nausea, headaches, spatial disorientation and vomiting [24].

## Methods/design

### Study site and population

The study is being conducted at La Conception University Hospital (Marseille, France). The inclusion criteria are as follows: subject 18 years old or older; subject with a past diagnosis of chronic smoking as defined by the DSM-V and with the presence of at least three of the 11 criteria in the DSM-V for nicotine dependence [25]; subjects reporting smoking abstinence for at least 1 week (defined by the total absence of tobacco consumption reported by the individual) and carbon monoxide levels expired less than 3 parts per million (ppm); subjects able to speak and read French; and subjects with a signed consent form.

The exclusion criteria are as follows: subjects younger than 18 years old; pregnant or breastfeeding individuals; subjects not covered by French national health insurance; subjects with decompensated organic and psychiatric disease; subjects with contraindications to virtual reality therapy, such as epilepsy or severe myopia (> −3.5 dioptres); subjects with dementia defined by a Mattis scale score < 136 [26]; subjects without a signed information and consent form or, for guardianship, subjects for whom a legal representative did not sign this form.

### Study design and procedure

The present study is a 14-month (2 months of therapy and 12 months of follow-up), prospective, comparative, randomized and open clinical trial, involving two parallel groups (CBT coupled with VRET versus CBT alone). Recruitment is based on local advertisements and media, such as newspapers, radio and television. A psychologist screens the subjects for eligibility. Diagnoses of past chronic smoking and nicotine dependence are based on the DSM-V [25], and the Mini International Neuropsychiatric Interview (MINI) French Version [27] is used to evaluate psychiatric comorbidities. Then, a physician describes the study, responds to any questions the candidates might have and obtains written informed consent. The participants are then randomly assigned to two therapeutic groups: one group receiving CBT coupled with VRET and one group receiving CBT alone. The allocation to each group is accomplished using a randomization table generated by a computerized sequence generator (1:1 allocation ratio). Data are collected during face-to-face interviews conducted by psychologists and also using self-reported measurements at five different time points: at randomization (baseline; T0), at the end of therapy (2 months, T1) and then at 3 (T2), 6 (T3) and 12 months (T4) after the end of therapy (see Table 1). Only one therapist is in charge of the sessions in the two groups.

**Table 1:** Assessment schedule

	T0 (Baseline)	T1 (2 months after T0)	T2 (3 months after T1)	T3 (6 months after T1)	T4 (12 months after T1)
Mattis^1^	X				
MINI^2^	X				
DSM-V nicotine dependence^3^	X	X	X	X	X
CDS-12^4^	X	X	X	X	X
FTCQ-12^5^	X	X	X	X	X
STAI-A^6^	X	X	X	X	X
BDI^7^	X	X	X	X	X
SF-12^8^	X	X	X	X	X
EES-10^9^	X	X	X	X	X
Co^10^	X	X	X	X	X

^1^Global Assessment Scale cognitive functions; ^2^Mini International Neuropsychiatric Interview French Version; ^3^Diagnostic and Statistical Manual of Mental Disorders, 5^th^ ed.; ^4^Cigarette Dependence Scale; ^5^Tobacco Craving Questionnaire; ^6^State-Trait Anxiety Inventory form A; ^7^Beck Depression Inventory; ^8^the SF-12 quality of life questionnaire; ^9^Rosenberg questionnaire: self-esteem scale; ^10^carbon monoxide levels expired

### Treatment groups

The protocol includes eight weekly individual therapeutic sessions of 60 minutes in duration for both groups. The eight sessions are conducted by psychologists with training in cognitive behavioural approaches and virtual reality exposure. The content of the CBT is similar in the two groups but delivered differently between the two groups.

1. CBT treatment group
  The subjects receive eight weekly individual sessions of CBT. The CBT was developed by three psychologists (CG, NP and MFP) with training in the assessment and treatment of health problems and specific training in CBT. The content was first developed independently by two of the psychologists (CG and NP), based on international guidelines, the scientific literature on smoking cessation and relapse and their professional experience [4, 7, 26, 27]. The sessions pivoted on all of the key principles of CBT. It was collaborative, present-oriented, and problem-focused. Any discrepancies were resolved by consensus with a third psychologist (MFP). The CBT was then tested on a small sample of patients (n = 10), confirming its acceptability and validity. The sessions were designed to target the factors linked to smoking relapse after smoking cessation, including: 1) definition of relapse and identification of causes leading to relapse; 2) analysis and detection of high risk situations; 3) explanation of automatic thoughts and beliefs leading to tobacco consumption; 4) presentation of strategies (for example, behavioural coping strategies and cognitive coping strategies); 5) management of negative emotions; 6) management of assertiveness. The sessions are described in Additional file 1.
2. CBT coupled with VRET treatment group
  The subjects receive two weekly individual sessions of CBT, followed by six weekly individual sessions of VRET. VRET consists of exposing the patient to situations considered to incur high risk of tobacco relapse and aimed at the reduction of cue reactivity by extinction. The therapist can help the subject to review the CBT principles and strategies during the VR exposure, using various complex environments tailored to the project (detailed below). The VR exposure is context graded because the smoking cues can vary in intensity (for example, number of avatars smoking around the patient, presence of cigarette boxes spread on tables). Given individual differences in the relevance of craving-specific stress among participants and to determine the order of exposure to virtual situations, a hierarchy of virtual environments (VEs), from the least craving and anxiety induced to the most craving and anxiety induced, was established for each participant independently. Throughout the exposure to VEs, the participants were invited to progress to the next VE when they had reached a comfortable level of emotion or craving in the current environment. Consequently, if required, exposure to a particular event or situation could be repeated. The therapist could monitor the subject's reactions according to the displayed situation. The session occurring in VEs lasts approximately 50 to 60 minutes. This duration is standard and does not depend directly on extinction or craving reduction. However the 10-minute lapse allows the therapist to ensure that the end of exposure take place when arousal or craving is lowered, thus avoiding any accidental sensitization phenomenon. Besides, when extinction and craving reduction do take place before minute 50, participants are invited to proceed to the next context graded VE [28, 29].

### Apparatus

The VR system includes a ruggedized Sensics ZSight HMD (1280x2024 stereoscopic OLED screen with 60° field of view), coupled with an embedded 3 degrees of freedom head tracker (angular resolution: 0,02°, latency 4 ms). The head tracker enables the subject to visually explore the environment by updating the 3D scene as a function of head orientation. Otherwise, the navigation is triggered by mouse motion. The subject's direction of locomotion is defined by his or her head orientation in the VEs. The participants must use a wireless controller with a directional pad for the walking locomotion. The steering wheel exploited for the driving VE is a Logitech G25 with vibration and force feedback capabilities. The VEs are generated and run on an ordinary graphics orientated notebook with a 4 core processor, 16Go DDR2 RAM, a graphics card with 3 Go RAM and a 1440x900 resolution screen. The required software is Microsoft Windows 7 (32- or 64-bit edition), Microsoft DirectX 9.0 or higher and the equipment’s drivers.

The heart rate monitor is a Polar RS800CX. It includes a transmitter and a wrist receiver. This monitor has proved to be as effective as an ECG for recording RR intervals and heart rate measurements [30].

### Software and virtual environments

The main software exploited to create and run the VEs was Sandbox. Sandbox is an inexpensive (30 to 60 USD) and commercially available game level editor (GLE) of the video game Crysis 1/2, exploiting the CryEngine game engine developed by Crytek GmbH. Prior to its full use for the trial, this GLE was tested and compared to seven other commercially available GLEs by considering several distinct criteria and requirements previously reported [31].

To construct the VEs, the experimenter exploited the aforementioned GLE to build six specific cue-graded VEs related to smoking. The VEs were selected to represent common situations in daily life involving high risk situations in terms of smoking relapse [32, 33]. These six VEs offered distinct craving-inducing scenarios:

1. Having a drink with friends smoking in a virtual bar at dusk;
2. Have dinner with avatars smoking on the terrace of a restaurant;
3. Having coffee after dinner at home;
4. Waiting at a bus stop with avatars smoking;
5. Taking a break in the workplace or studying with colleagues who are smokers; and
6. Driving a virtual car on a road with traffic.

**Fig. 1:**
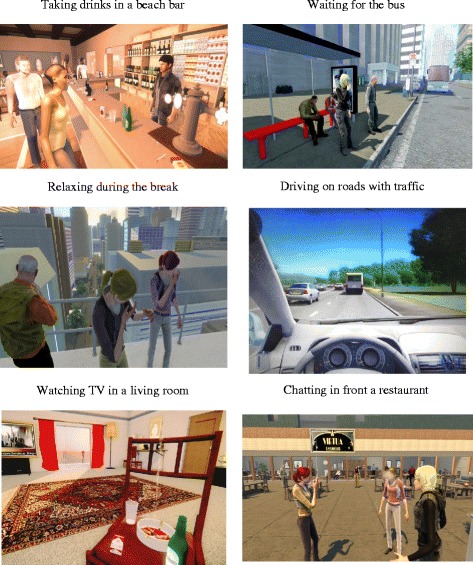
Screenshots of the virtual environments utilized in the present study. Note the surrounding avatars smoking cigarettes

The six VEs are presented in Fig. 1.

During the exposure, the experimenter can trigger specific events within the VE (for example, avatars talking about smoking or inviting the participants to smoke a cigarette). These options allow for increases in the intensity of induced craving to modulate the degree of exposure at various times. The dynamic VEs also provide the participant with direct, realistic interactions (such as doors, responding virtual humans, grabbing of objects and physical or mechanical reactions to the user’s presence).

The environments were validated in a small number of patients (n = 10) using self-reported subjective craving and psychophysiological measurements, in accordance with previous works on this issue [34].

The environments were created with respect to the EULA (end user license agreement): they can be shared on request and used solely for that purpose and not to generate profit.

### Evaluation outcome

1. Primary outcome
  The primary outcome is the proportion of individuals with tobacco abstinence at 6 months after the end of the therapy (T3) [35, 36]. Abstinence is defined by the total absence of tobacco consumption as assessed during a post-treatment interview with the participants about any possible relapses and an apparatus that measures the carbon monoxide levels expired for a more objective evaluation. If the results are greater than or equal to 3 ppm, the subject is considered to have relapsed [37, 38].
2. Secondary outcomes
  Tobacco abstinence is assessed using the carbon monoxide levels expired at T0, at the end of the therapy (2 months, T1) and at 3 (T2), 6 (T3) and 12 months (T4) after the end of therapy T2, T3 and T4.
  Tobacco dependence is assessed at T0, T1, T2, T3 and T4 with the DSM-V criteria and the Cigarette Dependence Scale (CDS-12) [39], a 12-item self-report instrument with scores ranging from 12 (no dependence) to 60 (high dependency). This scale has high test-retest reliability (≥0.83) and high internal consistency (Cronbach's alpha ≥0.84).
  Craving is assessed using two different measures. Smoking craving is evaluated at T0, T1, T2, T3 and T4 with the French Tobacco Craving Questionnaire (FTCQ-12) [40], a 12-item self-report instrument with scores ranging from 12 (no craving) to 84 (high craving). The internal consistency alpha coefficients were 0.83, 0.79, 0.69 and 0.66 for the different factors (emotionality, expectancy, compulsivity and purposefulness). Craving is also evaluated using an analogical craving scale, ranging from 0 (lack of desire) to 10 (extreme desire) [41] before, during and after each session.
  Anxiety is assessed at T0, T1, T2, T3 and T4 with the State-Trait Anxiety Inventory (STAI) [42] Y-A (state). The STAI is a 20-item self-report instrument with scores ranging from 20 (absence of anxiety) to 80 (high anxiety). The STAI is among the most widely researched and widely used measurements of general anxiety, with satisfactory internal consistency alpha coefficients (≥0.7).
  Depression is assessed at T0, T1, T2, T3 and T4 with the Beck Depression Inventory (BDI). The BDI is a 13-item self-report instrument. A total score between 4 and 7 shows a mild state of depression, between 8 and 15 an average to moderate state of depression, and 16 or higher a severe state of depression [43, 44]. This scale has satisfactory psychometric properties. A meta-analysis of the BDI's internal consistency estimates yielded a mean alpha coefficient of 0.81. The concurrent validity of the BDI with respect to clinical ratings and the Hamilton Psychiatric Rating Scale for Depression were also high. The BDI also distinguishes subtypes of depression and differentiates depression from anxiety [45].
  Quality of life is assessed at T0, T1, T2, T3 and T4 with the SF-12 [46], assessing physical function, physical pain, general health, vitality (energy and tiredness), social functioning and well-being, as well as limitations due to physical and mental health. Two composite scores are obtained with this self-report instrument: a Physical Component Score (PCS) and a Mental Component Score (MCS). A high score indicates a high level quality of life. Several studies have reported that the SF-12 is able to produce the two summary scales originally developed from the SF-36, one of the most widely used quality of life instruments, with considerable accuracy and yet with far less of a respondent burden [46, 47].
  Self-esteem is assessed at T0, T1, T2, T3 and T4 with the Rosenberg questionnaire [48]. This self-report questionnaire consists of 10 items and produces a total score of 10 to 40; a high score indicates high self-esteem. This questionnaire is a reliable and valid measurement of global self-worth [49].

The following data are collected at each session (see Table 2).

**Table 2:** Assessment during the eight therapy sessions

	Cravings at the beginning of the session	Cravings during the session	Cravings at the end of the session	HR^1^	HRV^2^	PQ^3^	SSQ^4^
CBT	X		X				
CBT coupled with VRET	X	X	X	X	X	X	X

^1^Heart rate; ^2^heart rate variability; ^3^Presence questionnaire; ^4^Simulation Sickness Questionnaire

HR and HRV are measured during the VRET session

PQ and SSQ are measured at the end of the VRET session

- Perception of presence is assessed by the Presence Questionnaire (PQ), version 3.0 [50]. The PQ consists of 32 items rated on a 7-point scale, and factor analyses found 6 factors: involvement, interface quality, adaptation and immersion, consistency with expectation, visual fidelity and auditory fidelity [51]. The PQ is a self-report instrument that has been validated in many empirical studies [50–53] and has also demonstrated a relationship between presence and anxiety [52]. A high score indicates a satisfactory perception of presence.
- Symptoms reported to be associated with simulator sickness are assessed with the Simulation Sickness Questionnaire (SSQ) [53]. The SSQ is a 16-item self-report instrument with scores ranging from 16 (absence of cybersickness) to 48 (high cybersickness). Factor analyses found three main factors: oculomotor (for example, blurred vision), disorientation (for example, dizziness) and nausea (for example, vomiting).
- *Heart rate* (HR) and *heart rate variability* (HRV) are evaluated with a heart rate monitor (apparatus for technical description) for the duration of each therapeutic session. The HR reflects the average heart rate recorded during the therapeutic session. The HRV indicates the fluctuations in heart rate around an average heart rate [30]. HR and HRV reflect the autonomic responses involved in emotional arousal, most notably during anxiety, during which the HR is expected to increase and the HRV to decrease [54]. There is evidence that heart rate changes are correlated with presence [55]. Furthermore, HR is one of the main correlates of craving [8]. HR and HRV represent alternative objective measurements of anxiety response, presence and craving.

### Sample size

The sample size was calculated [56] to obtain 80 % power to detect a 30 % difference between the two groups in the proportion of individuals with tobacco abstinence at 6 months (reference point = 30 % [36]). With a significant *P*-value of 0.05, these calculations showed that a total of 49 individuals per group was required, allowing for, with 15 % of patients potentially being lost to follow-up, a total of 120 needing to be included.

### Statistical analysis

The data will be summarized using the means, medians, standard deviations and ranges for quantitative data and counts and frequencies for categorical data. The analyses of the primary and secondary outcomes will be performed on the intent-to-treat population. We will conduct analyses for each of the outcomes separately. Complementary per protocol analyses will be performed (that is, comparisons of patients who completed the treatment originally allocated). Finally, missing data will be addressed when possible using multiple imputations. No interim analysis is planned.

Comparisons between the two groups for each outcome will be performed using Student’s *t*-tests or the Mann-Whitney-Wilcoxon test for quantitative or ordinal variables and the chi-square test or Fisher’s exact test for frequencies. Non-parametric tests will be used for data that are not normally distributed. Moreover, logistic regression models will be used. The dependent variable will be the primary outcome (that is, the rate of individuals with tobacco abstinence). Explanatory variables will be selected among those for whom the *P*-value is less than or equal to 0.20 in univariate analysis, and they have been described in the literature as being associated with tobacco abstinence and relapse.

Statistical significance is defined as *P* > 0.05. Statistical analyses will be performed using SPSS statistics software, version 17.0 for Windows (SPSS Inc., Chicago, IL, USA).

### Ethical principles and safety

The study is designed and conducted in accordance with the principles of the Declaration of Helsinki, seventh revision [57]. The patients are provided with both oral and written information regarding the study prior to obtaining their informed consent. The local ethics committee (CPP Sud Méditérannée V) approved this study, which is registered with the international standard randomized controlled trial number (NCT01570712).

## Discussion

The present study aims to assess the effectiveness of CBT coupled with VRET in the prevention of smoking relapse in subjects with smoking abstinence. VRET in association with CBT might represent an interesting alternative to CET. Several previous studies have supported the efficacy of VRET for smoking cessation [17–19]. VR environments can be used effectively in the treatment of nicotine addicts who wish to give up smoking [32]. We hypothesize that the findings of our study might be in agreement with their findings. From a theoretical perspective, CET appears to be a relevant method for complementing CBT according to the conditioning model [58]. It focuses on prompting the patient to contend with smoking-related stimuli (for example, the tobacco package, ashtrays, lighters, cigarettes) to mitigate smoking behavioural responses, to decrease withdrawal complications and to extinguish the craving. However, CET has an important limitation. Smoking-related stimuli are presented only in proximal confrontation patterns, such as images, photos and videos (that is, proximal risks), so there is no interaction with the environment or its multi-sensorial stimuli (distal risks), which are known to be of the utmost importance in smoking relapse. VRET might thus offer an alternative line of treatment by presenting the simultaneous presence of proximal and distal stimuli, compared to CET. Our hypothesis was that individuals would learn with VRET how to cope with proximal and distal risks and then use effective skills when facing similar environments *in vivo*.

The effects of VRET on health outcomes, such as anxiety, depression and quality of life, constitute an original aspect of our study, compared with previous studies. Several studies have reported that negative affect plays an important role in maintaining smoking dependence [21]. Our hypothesis was that VRET might have an indirect effect, mediated by the impact on smoking dependence, on negative affect (depression) and other psychological features, such as anxiety, self-esteem and quality of life.

The last objective is to demonstrate that this novel virtual environment is feasible and acceptable to the clinicians and patients who are potential program users. This objective is of the utmost importance, because the successful application of any technology as a tool to enhance evidence-based treatments is directly related to the ability of patients and clinicians to use the tool easily and effectively.

Several limitations must be borne in mind when considering our protocol. First, we chose to propose a widely used design for the CBT intervention, based on eight weekly individual therapeutic sessions of 60 minutes in duration. Because CBT is already a constraining treatment, we could not propose performing VRET in addition to CBT sessions of 60 minutes in duration. To keep the same number of sessions between the two groups, we proposed to the VRET treatment group two full sessions dedicated CBT (that is, concentrating the eight sessions of the CBT treatment group), followed by six sessions for VR exposure. This choice might mitigate the full integration of CBT and VRET and thus decrease the effectiveness of their associations. The differences in group conditions will be examined with greater attention when interpreting the results of this study. In addition, this choice might also decrease the effectiveness of CBT in the group with VRET because the principles cannot be learned as rapidly (that is, in two sessions) by patients. However, the therapist can help the participants during the VRET sessions to review the principles of CBT once they are immersed in the virtual environments. Second, the virtual environments used in our trial are situations featuring only pleasant connotations (such as being in a restaurant or on the beach). A whole gamut of cues with negative connotations (such as job stress, arguing with one’s spouse, acute stress, receiving bad news) that can trigger tobacco cravings exists as well in reality. In fact, former smokers might be more sensitive to cues with negative emotional valences and more prone to relapse in this context, rather than during positive events. Future studies focusing on situations with negative connotations should be performed. Finally, heart rate (HR) was examined only in the CBT coupled with VRET treatment group and not in the CBT treatment group alone. Because the CBT treatment group did not include cue exposure therapy, we did not include a measurement of HR data. Indeed, HR could be considered physiological reactivity to smoking-related cues, and it was measured during the cue presentations (VRET session). Our hypothesis was that VRET lowers physiological reactivity to smoking-related cues and that we would observe a significant decrease in HR during the VRET sessions. We expect to observe a negative correlation between HR and the number of VR sessions. The absence of comparisons for this parameter between the two groups is a limitation of our study. However, HR is one of the main correlates of craving, and we compared smoking cravings between the two groups with the French Tobacco Craving Questionnaire (FTCQ-12).

Finally, the findings of our study might have significant implications for future population-based interventions. Because VRET is now available at a low cost and is simple to use [19], this interactive therapeutic method might be easily implemented in clinical practice if our study confirms its efficacy. We can also hypothesize that, in the long run, patients will practice VR exposure at home. Advances in computer science, hardware performance and equipment availability make this assumption entirely probable. Events such as the acquisition of the Oculus company, which assembles affordable VR equipment, by Facebook or the use of ordinary Samsung cell phones as screens for HMD Gear VR could represent premises for the generalization of VRET in the general population and could induce a major shift in the media paradigm in post-modern societies.

### Trial status

The study started including participants in August 2014, and the recruitment is ongoing.

## Additional file

Additional file 1:
**Description of CBT sessions.** (DOCX 40 kb)

## Abbreviations

BDI: Beck Depression Inventory
CBT: cognitive behavioural therapy
CDS-12: Cigarette Dependence Scale
CET: cue exposure therapy
DSM-V: Diagnostic and Statistical Manual of Mental Disorders, 5th version
EULA: end user license agreement
FTCQ-12: French Tobacco Craving Questionnaire
GLE: game level editor
HR: heart rate
HRV: heart rate variability
PQ: Presence Questionnaire
SF-12: Short Form Health Survey
SSQ: Simulation Sickness Questionnaire
STAI: State-Trait Anxiety Inventory
VE: virtual environment
VR: virtual reality
VRET: virtual reality exposure therapy
