# Supplementary material for: Virtual reality cue exposure for the relapse prevention of tobacco consumption: a study protocol for a randomized controlled trial
Source: Trials. 2016 Feb 19;17:96. doi: 10.1186/s13063-016-1224-5 (PMC4759851; doi:10.1186/s13063-016-1224-5)
Supplement: Additional file 1: — Description of CBT sessions. (DOCX 40 kb) [file 13063_2016_1224_MOESM1_ESM.docx]

Additional file 1. CBT Sessions.

- Session 1 and 2:

Aim: to define «relapse» and identify causes leading to relapse (session 1) ; to analyze high risk situations and relapse (What are the feelings/thoughts before, during and after the relapse) (session 2).

- "What is a relapse for you? Have you ever experienced a relapse? Do you think you could live a relapse in future and what would be the events, thoughts, emotions that might cause a relapse? If you have a relapse, what would be your reaction? Do you know any strategies that might allow you to recover from a relapse? If yes, which ones? According to you, how can we avoid the risk of relapse?".

-Question and dialogue centered on situation, feelings and thoughts: "when, where, how and with whom took place the relapse? What are the feelings felt before, during and after the relapse? Are they positive or negative? In what do they favor the relapse? What were the thoughts before, during and after the relapse? In what do these thoughts prompt the relapse?".

- Session 3 and 4

Aim: to explain automatic thoughts/beliefs leading to the tobacco consumption (session 3); presentation strategies (behavioral coping strategies; cognitive coping strategies) (session 4).

- Work on different thoughts, on automatic thoughts/beliefs leading to the tobacco consumption. Defining each thought and explains its content.

- Presentation/unveiling strategies: initial strategies to contend with temptations; behavioral coping strategies; cognitive coping strategies. Defining and explaining the suggested methods.

- Session 5 and 6

Aim: management of negative emotions.

- What is an emotion? Why work on negative emotions? How negative emotions can lead to relapse? Being aware of negative thoughts. Managing negative thoughts.

- Session 7

Aim: assertiveness (i.e., how to refuse and How to consider criticism).

- Definition of assertiveness.
- Presentation of techniques/methods to refuse and to receive criticism.
- Role Play to apply techniques/methods described earlier in the session.

- Session 8

Aim: assessment, feedback and follow-up

- Summary of the previous sessions, notions that have been learned, methods that has been understood. Going back over past misconceptions or unclarified principles. Reviewing what was efficient or inefficient to maintain the for the subject abstinence.

- Reviewing the various pitfalls involved in tobacco (relief printing, illusion of relaxation, hope to lose weight).

- Stressing the role of high risk smoking related situations with the subject and reviewing erroneous beliefs in order to ensure that these can be recognized. Demonstrating the influence of smoking in the subject's inner speech about their decision to smoke. Being aware of the chain of behaviors connected with chronic smoking and seeking alternative strategies to this behavior.
